# Complete Genome Sequencing of Bovine Viral Diarrhea Virus 1, Subgenotypes 1n and 1o

**DOI:** 10.1128/genomeA.01744-15

**Published:** 2016-02-18

**Authors:** Asuka Sato, Kentaro Tateishi, Minami Shinohara, Yuki Naoi, Mai Shiokawa, Hiroshi Aoki, Keitaro Ohmori, Tetsuya Mizutani, Junsuke Shirai, Makoto Nagai

**Affiliations:** a Cooperative Department of Veterinary Medicine, Faculty of Agriculture, Tokyo University of Agriculture and Technology, Fuchu, Tokyo, Japan; b Research and Education Center for Prevention of Global Infectious Disease of Animals, Tokyo University of Agriculture and Technology, Fuchu, Tokyo, Japan; c Faculty of Veterinary Science, Nippon Veterinary and Life Science University, Musashino, Tokyo, Japan

## Abstract

To gain further insight into the genomic features of bovine viral diarrhea virus 1 (BVDV-1) subgenotypes, we sequenced the complete genome of BVDV-1n Shitara/02/06 and BVDV-1o IS26NCP/01. The complete genome of Shitara/02/06 and IS26NCP/01 shared 77.7 to 79.3% and 78.0 to 85.7% sequence identities with other BVDV-1 subgenotype strains, respectively.

## GENOME ANNOUNCEMENT

Bovine viral diarrhea virus (BVDV), classic swine fever virus, and border disease virus belong to the genus *Pestivirus*. BVDV is a member of the family *Flaviviridae* and is divided into two species, BVDV-1 and BVDV-2 (1, 2). BVDV is an economically important pathogen of livestock, and infects not only bovine but also swine and small ruminants (1, 2). Currently, BVDV-1 is subdivided into at least 17 subgenotypes, BVDV-1a to -1q (3–8). Further insight into the genomic features of these subgenotypes is important in order to have a more comprehensive understanding of pestiviruses. BVDV-1n and BVDV-1o are rare subgenotypes that have only been identified in Japan, South Korea, and China (4, 6, 9, 10). BVDV-1n strain Shitara/02/06 and BVDV-1o strain IS26NCP/01 were isolated from calves that were persistently infected with BVDV in Japan in 2006 and 2001, respectively (6). Although partial sequences of these strains were reported, whole-genome sequences of them were not determined. In this study, we sequenced the complete genomes of Shitara/02/06 and IS26NCP/01.

Viral RNA was extracted from the supernatant of virus-infected Madin-Darby bovine kidney cells using TRIzol LS reagent (Life Technologies, Carlsbad, CA, USA), followed by treatment of the RNA with DNase I (TaKaRa Bio Inc., Shiga, Japan). The cDNA libraries were constructed using the NEBNext Ultra RNA library prep kit for Illumina version 2.0 (New England Biolabs, Ipswich, MA, USA), as described previously (11). The libraries were loaded onto a MiSeq cartridge and sequenced using a MiSeq benchtop sequencer (Illumina, San Diego, CA, USA). Sequence reads were assembled into contigs by the *de novo* assembly algorithm from the CLC Genomics Workbench 6.5.1 (CLC bio, Aarhus, Denmark). The 5′ and 3′ genome sequences were determined using the rapid amplification of cDNA end (RACE) method (5′-full RACE core set and 3′-full RACE core set; TaKaRa Bio, Otsu, Japan). The whole-genome sequences of Shitara/02/06 and IS26NCP/01 comprised 12,266 and 12,268 nucleotides (nt), including 387 nt and 386 nt long 5′ untranscribed regions (UTR), and 182 nt and 185 nt long 3′ UTR, and included an open reading frame (ORF) that encompassed 3,898 and 3,898 amino acids (aa), respectively. No insertion of cellular genomic sequences or duplication of viral genomic sequences was identified in their genomes.

The sequences of Shitara/02/06 and IS26NCP/01 were compared with other BVDV-1 subgenotype strains in the database. While most of the BVDV-1 subgenotype strains shared <80% nt identity with the whole-genome sequences of Shitara/02/06 (77.7 to 79.3%) and IS26NCP/01(78.0 to 79.3%), there were some notable exceptions: IS26NCP/01 compared to BVDV-1m ZM-95 (85.7%) and IS26NCP/01 compared to BVDV-1q Camel-6 (81.5%). In addition, Shitara/02/06 and IS26NCP/01 shared 69.7 to 70.0% and 69.5% identity with BVDV-2 strains, and 67.5% and 67.3% identity with BVDV-3 D32/00_HoBi, respectively. This study presents the first report of the whole-genome sequences of BVDV-1n and -1o, and may provide further insight into the genomic features of BVDV-1 subgenotypes.

### Nucleotide sequence accession numbers.

The full genomic sequences of IS26NCP/01 and Shitara/02/06 were deposited in the DNA Data Bank of Japan and DDBJ/EMBL/GenBank database under the accession numbers LC089875 and LC089876, respectively.
